# Importance of swift event adjudication of endpoints for adequate reporting to data and safety monitoring boards in clinical trials—lessons from CULPRIT-SHOCK

**DOI:** 10.1186/s13063-021-05129-4

**Published:** 2021-03-08

**Authors:** Peter Clemmensen, Benedikt Schrage, Uwe Zeymer, Holger Thiele, Karl Wegscheider

**Affiliations:** 1 University Heart and Vascular Center Hamburg, Department of Cardiology, Hamburg, Germany; 2 Klinikum Ludwigshafen, Ludwigshafen am Rhein, Germany; 3 Heart Center Leipzig at University of Leipzig and Leipzig Heart Institute, Leipzig, Germany; 4 University Hospital Hamburg Eppendorf, Hamburg, Germany

Conduction of randomized controlled clinical trials is cost- and time-consuming, especially if study enrolment is performed at multiple sites in multiple countries with different medical traditions, medical record systems, and language barriers. Typically, three distinct boards are created to coordinate these trials. First of all, the principal investigator and associated advisors form the *Steering Committee*, which is responsible for executive tasks such as trial design and oversight. Secondly, a *Data Safety Monitoring Board* (DSMB) is needed to review the event and outcome data. This board consists of independent members and is responsible for ongoing evaluation of participant safety, overall conduction as well as the progress of the trial and might even recommend to modify or terminate the trial in case of respective concerns. Thirdly, the *Clinical Event Adjudication Committee* (CEAC) reviews the anonymized participant data and adjudicates events and outcome data. While the DSMB has direct access to the random code, the CEAC remains blinded throughout the trial to guarantee unbiased adjudications. Whereas the latter is often considered a necessity for the final reporting and thus robustness of the conclusions it is also quite common that substantial delays occur between investigator reporting and final adjudication of events. There are practical and financial interests to have fewer CEAC gatherings. While this delay is commonly accepted by the steering committee as it occurs simultaneously with other activities before the data lock of a trial, this delay may have important consequences for the interpretation during interim analyses, i.e. for the DSMB. Cardiovascular outcomes trials often include interventions which could benefit patients but inherently also might incur harm. In recent years there have been several examples of clinical trials being stopped prematurely due to either benefit or risk, even with an unfavorable risk-benefit ratio [1–3]. Increasingly, clinical trials have endpoints which are a composite of two or more adverse outcomes with quite different pathophysiological mechanisms or end organ involvement. As a consequence, the particular endpoints in a clinical trial can trend in quite opposite directions, making the interpretation difficult.

An example of possible causes of death trending in different directions was encountered during the conduction of the recently published CULPRIT-SHOCK trial [4]. This trial was the first large randomized trial which proved that the hitherto preferred strategy of immediate complete revascularization with PCI in cardiogenic shock led to more harm defined by the primary endpoint of death and need for renal replacement therapy with an absolute difference of 9.5%. These 30-day results were recently confirmed in the 12-month follow-up analysis [5]. The pre-specified interim analysis after inclusion of 342 patients presented to the DSMB already showed a significant difference in 30-day total mortality favoring culprit-lesion only versus immediate multivessel PCI (44.2% vs. 57.1%; *p* = 0.017). The primary outcome of the trial, which also included renal replacement therapy, displayed a similar difference (47.1% vs. 61.2%; *p* = 0.009). The protocol stated that the interim analysis should be performed according to the O’Brian-Fleming method. The trial was to be stopped “if the null hypothesis of equal event rates can be rejected with a significance level of 0.005”. Thus, the attained *p* value for the primary endpoint at the time when half the patients had been enrolled was close to the stopping rule with a 0.004 margin. Although not formally reaching the pre-specified boundary, the difference in total mortality was of concern to the DSMB. The main reason for not recommending a termination of the trial for safety reasons at this time point were mixed signals in the causes of death, possibly attributed to the fact that independent endpoint adjudication was lagging behind. The most common investigator reported cause of death, “refractory cardiogenic shock” was evenly distributed in the entire cohort: 49/170 in the multivessel PCI arm (28.8%) and 52/172 (30.2%) in the culprit only arm. However, among the fatal cases, “refractory cardiogenic shock” was significantly less common with multivessel PCI (50.5% (49/97) vs. 68.4% (52/76) of total death, *p* = 0.018) in line with the previous pathophysiological mainstream thinking that multivessel PCI would serve as a guardian against myocardial ischemia, re-infarctions and thus prevention of shock progression. In support of this possible pathophysiological mechanism, there was a numerically lower rate of deaths from re-infarctions in the multivessel PCI arm (0% (0/97) vs. 2.6% (2/76); *p* = 0.11). Furthermore, in this phase of the trial with incomplete adjudication, there was an imbalance in the deaths attributed to “other” or “unknown” causes. The increase in total mortality in the immediate multivessel PCI arm resulted from an excess in “unknown” (5.2% (5/97) vs. 0% (0/76); *p* = 0.05) and “other causes” of death (20.6% (20/97) vs. 11.8% (9/76); *p* = 0.13). The aforementioned values are depicted in Fig. 1.

**Fig. 1:**
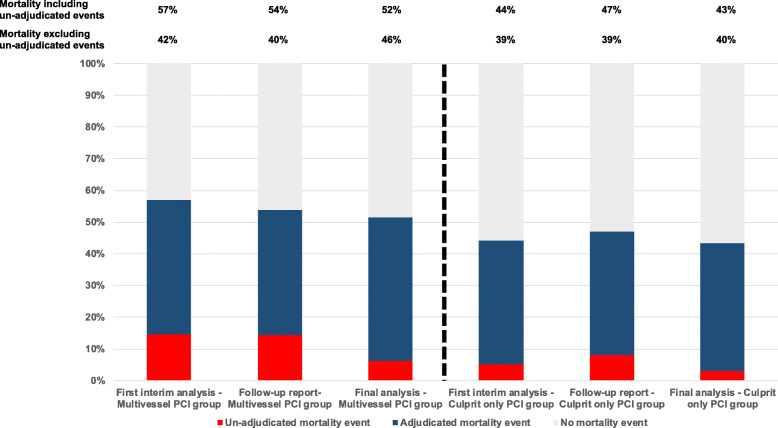
Comparison of adjudicated and un-adjudicated mortality events during the life cycle of the CULPRIT-SHOCK study, as reported to the DSMB

These different trends made it difficult to assess whether there were inacceptable risks for the study population which prompted the DSMB to address the steering committee and recommend a continuation of the study under the condition that an immediate plan be set forward for the CEAC to promptly adjudicate the reported events. During this endeavor, to provide an updated adjudication of causes of death report to the DSMB, inclusion in the trial also picked up. In the follow-up report which included 676 patients, the absolute difference in total 30-day mortality decreased from 13% to 7% and was no longer significant. Also, the primary endpoint was not significantly different between the groups at this timepoint (61.4 vs 53.8%; *p* = 0.07). Death from refractory cardiogenic shock however remained, albeit less pronounced, numerically in favor of multivessel PCI (49.7 vs 62.9%). At this point, the DSMB advised that the study be completed as originally set out, and the DSMB saw no convincing argument to stop the trial for safety reasons. With respect to CULPRIT-SHOCK, the DSMB did not perform a second interim analysis and was not concerned about different trends in the two components of the primary endpoint, but about different trends in the causes of death which can be understood as components of the most important safety variable, mortality. The thorough discussion of the safety risks after 676 patients were included was not a second interim analysis and was not restricted to the primary endpoint. Ultimately, the percentage of un-adjudicated mortality events decreased and was comparable between both study arms while the finding of a superiority of the culprit-lesion-only PCI strategy prevailed.

Stopping a trial prematurely always leads to discussions regarding the appropriateness. Often very spectacular differences early in a trial tend to show some regression toward the mean with time and more patients included, as observed in CULPRIT-SHOCK. Furthermore, one should be particularly careful when stopping relatively small studies in complex patients, where a second pivotal trial is unlikely to emerge to guide physicians, authorities, and ultimately treatment guidelines. A formal stopping rule should in such cases have the narrowest possible confidence interval. In the case of CULPRIT-SHOCK the DSMB was also aware of the difficulties in attributing a cause of death in patients with multiorgan failure, and thus competing causes of death, with most patients dying within 48 h of admission.

With this letter, we want to stress the importance of reliable and fast event adjudication to ensure adequate interpretation of data for the safety of participants and proper reporting between boards in all clinical trials, not only cardiology. Every DSMB wants the particular trial to succeed but also has to remain strict and keep patient safety as the primary goal. The discussion remains open whether DSMB’s are bound by stopping rules or merely guided, especially in trials where clinical adverse events may trend in different directions.

## Data Availability

Not applicable.
